# Analyses of a Ceftazidime-Avibactam-Resistant *Citrobacter freundii* Isolate Carrying *bla*_KPC-2_ Reveals a Heterogenous Population and Reversible Genotype

**DOI:** 10.1128/mSphere.00408-18

**Published:** 2018-09-26

**Authors:** Mariana Castanheira, S. J. Ryan Arends, Andrew P. Davis, Leah N. Woosley, Amira A. Bhalodi, Shawn H. MacVane

**Affiliations:** a JMI Laboratories, North Liberty, Iowa, USA; b Allergan plc, Irvine, California, USA; c Department of Pharmacy Services, Medical University of South Carolina, Charleston, South Carolina, USA; d Division of Infectious Diseases, College of Medicine, Medical University of South Carolina, Charleston, South Carolina, USA; Antimicrobial Development Specialists, LLC

**Keywords:** *Citrobacter freundii*, β-lactams, ceftazidime-avibactam, resistance mechanism

## Abstract

A *bla*_KPC-2_-carrying Citrobacter freundii isolate developed ceftazidime-avibactam resistance during treatment with this agent. The initial and follow-up isolates exhibited ceftazidime-avibactam MICs of 4 and 64 µg/ml, respectively. Overexpression of AcrAB-TolC and porin alterations were detected in both isolates, but no other resistance mechanism was observed. After passaging the initial clinical isolate in ceftazidime-avibactam at a fixed concentration of 4 µg/ml and a 4:1 ratio, resistance to all β-lactams was noted, and a percentage of the *bla*_KPC-2_ sequencing reads had mutations leading to the alterations D176Y (*bla*_KPC-2_-D176Y [78%]) or R164S plus P147L (*bla*_KPC-2_-R164S + P147L [82%]). Further investigation of the follow-up isolate showed that 11% of the *bla*_KPC-2_ reads had mutations leading to D179Y substitution (*bla*_KPC-2_-D179Y). In the absence of selective pressure, ceftazidime-avibactam MICs of the passaged and follow-up isolates revealed that 7 or 8 out of 20 screened colonies reverted to susceptible and possessed *bla*_KPC-2_ wild-type sequences. Recombinant plasmids carrying the *bla*_KPC-2_ alterations observed were transformed in Escherichia coli, and MIC values for ceftazidime ± avibactam were elevated. Lower MICs for ceftriaxone, cefepime, aztreonam, meropenem, and imipenem for the mutated KPC-2-producing isolates were observed compared to those of the isolates producing a wild-type KPC-2. Avibactam at a fixed concentration of 4 µg/ml restored the activity of all β-lactams tested for the recombinant strains. The heterogenous population of wild-type and mutated *bla*_KPC-2_ and the reversibility of the genotypes observed suggest a significant challenge for managing KPC-producing isolates that develop ceftazidime-avibactam resistance during therapy.

**IMPORTANCE** The development of ceftazidime-avibactam resistance among KPC-producing isolates during treatment with this agent has been reported. Usually isolates that become resistant have a mutated *bla*_KPC_ gene that confers resistance to ceftazidime-avibactam and susceptibility to meropenem. We report a Citrobacter freundii isolate that developed ceftazidime-avibactam resistance due to mutations within the coding region of the *bla*_KPC-2_ Ω-loop previously reported; however, in this case, only 11% of the whole-genome sequencing reads had mutations, making this alteration difficult to detect and the treatment of these isolates more challenging. In addition to *bla*_KPC_, the initial and the follow-up patient isolates displayed hyperexpression of the AcrAB-TolC efflux system and disruption of the outer membrane protein (OMP) OmpF, which contribute to carbapenem resistance. Experiments performed to confirm our findings included generating mutant isolates from the initial patient isolate, passaging the isolates for purity in drug-free medium, resulting in a reversible phenotype, and cloning the mutations to demonstrate the resistance conferred.

## Abstract (precis)

The development of ceftazidime-avibactam resistance among KPC-producing isolates during treatment with this agent has been reported. Usually isolates that become resistant have a mutated *bla*_KPC_ gene that confers resistance to ceftazidime-avibactam and susceptibility to meropenem. We report a Citrobacter freundii isolate that developed ceftazidime-avibactam resistance due to mutations within the coding region of the *bla*_KPC-2_ Ω-loop previously reported; however, in this case, only 11% of the whole-genome sequencing reads had mutations, making this alteration difficult to detect and the treatment of these isolates more challenging. In addition to *bla*_KPC_, the initial and the follow-up patient isolates displayed hyperexpression of the AcrAB-TolC efflux system and disruption of the outer membrane protein (OMP) OmpF, which contribute to carbapenem resistance. Experiments performed to confirm our findings included generating mutant isolates from the initial patient isolate, passaging the isolates for purity in drug-free medium, resulting in a reversible phenotype, and cloning the mutations to demonstrate the resistance conferred.

## INTRODUCTION

Isolates producing Klebsiella pneumoniae carbapenemase (KPC) enzymes are widespread in the United States and other countries. Until recently, the treatment options for organisms producing this carbapenemase were limited to antimicrobial agents that display toxicity issues or have a suboptimal distribution in certain infection sites or the scarce agents active against the organisms carrying these enzymes, which are often multidrug resistant.

Ceftazidime-avibactam has been approved in the United States since 2015, and this combination demonstrated good activity against isolates producing KPC enzymes and other serine β-lactamases. Ceftazidime-avibactam was superior to other regimens for treating carbapenem-resistant Klebsiella pneumoniae (1), Enterobacteriaceae (2), and carbapenemase-producing isolates (3) collected in U.S. hospitals.

Shortly after the introduction of this combination in clinical use, reports of isolates developing ceftazidime-avibactam resistance were published (4). The initial report was from a K. pneumoniae isolate producing KPC-3 that recently had been shown to have alterations in outer membrane proteins (OMPs) and hyperexpression of the AcrAB-TolC efflux system (5). Later studies demonstrated that mutations in the KPC-encoding gene leading to the D179Y substitution codified resistance to ceftazidime-avibactam (6). In most cases, the isolates displaying these mutations are susceptible to meropenem and the mutations described increase ceftazidime hydrolysis by creating a deeper pocket that traps the ceftazidime molecule (7, 8). After reading these reports, many hypothesized if ceftazidime-avibactam resistance developed during treatment that changing to meropenem would be an option.

In this study, we report a clinical case of ceftazidime-avibactam resistance developing in a KPC-2-producing Citrobacter freundii isolate during treatment with this combination and the characterization of the ceftazidime-avibactam-resistant isolate. Due to the initial lack of resistance mechanisms that could explain the resistance developed during therapy, we submitted the initial clinical isolates to passaging experiments in ceftazidime-avibactam, and we observed a heterogenous population of ceftazidime-avibactam-resistant *bla*_KPC-2_ mutants and wild-type (WT) sequences. Constructs were created to show the effect of each mutation on the susceptibility to β-lactams alone and in the presence of avibactam.

## RESULTS

### Clinical case.

**FIG 1:**
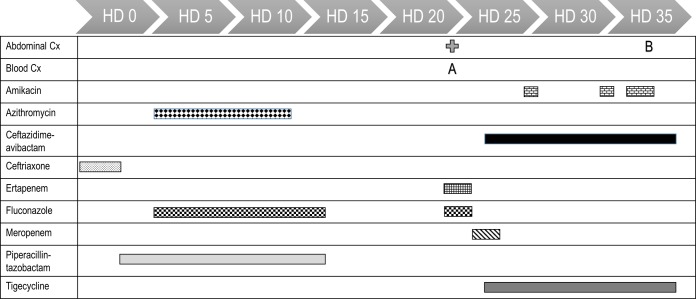
Infection and antibiotic timeline. The bars represent the duration of therapy for the antibiotics received. Abbreviations: A, initial Citrobacter freundii isolate from blood; B, follow-up Citrobacter freundii isolate from abdominal fluid; Cx, culture; HD, hospital day.

A 44-year-old woman with a failed renal transplant who was on peritoneal dialysis presented to a tertiary-care hospital in South Carolina in September 2015 with epigastric pain, nausea, and vomiting for 3 weeks. On hospital day 3 (HD 3), the patient was placed on piperacillin-tazobactam for a perforated duodenum. After multiple surgeries and washouts, the patient was no longer a candidate for operative intervention due to hemodynamic instability and was being managed conservatively with drainage. Piperacillin-tazobactam was discontinued on HD 17. On HD 24, a percutaneous left-upper-quadrant drain and a biliary drain were placed. On HD 26, blood cultures (initial isolate; Fig. 1) and abdominal fluid drain cultures were positive for C. freundii. The patient was started on ceftazidime-avibactam and tigecycline. The initial ceftazidime-avibactam dosing was 2.5 g every 12 h. The dose was increased to 2.5 g every 8 h on HD 33 based on the patient receiving aggressive continuous renal replacement therapy (CRRT) (9). Repeat blood cultures collected on HD 29 and HD 33 were negative, but the patient minimally improved from a clinical standpoint. Amikacin was added on HD 29. The infectious source was feculent peritonitis with signs and symptoms suggesting persistent intra-abdominal infection, but the patient was not a candidate for operative intervention due to hemodynamic instability. On HD 37, repeat abdominal fluid cultures grew C. freundii (follow-up isolate). The patient died on HD 39 due to multiorgan failure, untreatable intra-abdominal bleeding, coagulopathy, and liver failure.

### Characterization of C. freundii clinical isolates.

The initial C. freundii isolate and the follow-up isolate from the patient displayed MIC results within ±1 log_2_ dilution for most antimicrobial agents tested, except for ceftazidime-avibactam. The isolates were resistant to ceftazidime alone, cefepime, meropenem, piperacillin-tazobactam, aztreonam, levofloxacin, and tobramycin (Table 1). Both isolates were susceptible to amikacin and tigecycline when the Clinical and Laboratory Standards Institute (CLSI) or U.S. Food and Drug Administration breakpoints were applied, and colistin MIC values were wild type when the CLSI epidemiologic cutoff value was applied (10). The initial isolate displayed a ceftazidime-avibactam MIC of 4 µg/ml, and the follow-up isolate had an MIC of 64 µg/ml for this combination. The decreased ceftazidime-avibactam susceptibility of the follow-up isolate was confirmed by Etest (initial isolate MIC of 3 µg/ml and follow-up isolate MIC of 128 µg/ml) and disk diffusion (initial isolate zone diameter of 24 mm and follow-up isolate zone diameter of 16 mm). Pulsed-field gel electrophoresis analysis showed that the 2 C. freundii clinical isolates were indistinguishable (data not shown).

**TABLE 1:** Susceptibility profiles of *C. freundii* clinical isolates from the patient and after ceftazidime-avibactam passaging experiments

Antimicrobial agent(s)	MIC (µg/ml) of isolate^*a*^			
	Initial isolate from patient (*bla*_KPC-2_ WT)	Follow-up isolate (*bla*_KPC-2_-D179Y [11%])	Passaged on ceftazidime- avibactam at fixed concn of 4 µg/ml of inhibitor (*bla*_KPC-2_-D176Y [78%])	Passaged on ceftazidime- avibactam at 4:1 ratio (*bla*_KPC-2_-R164S + P174L [82%])
Ceftazidime-avibactam	4	64	32	64
Ceftazidime	512	>512	512	>512
Ceftriaxone	>64	>64	>64	>64
Cefepime	>64	>64	>64	64
Ampicillin-sulbactam	>32	>32	>32	>32
Piperacillin-tazobactam	>64	>64	>64	>64
Doripenem	>8	>8	>8	>8
Imipenem	32	16	8	8
Meropenem	64	32	32	16
Aztreonam	>16	>16	>16	>16
Doxycycline	4	4	4	8
Tetracycline	8	8	8	8
Ciprofloxacin	>4	>4	>4	>4
Levofloxacin	>4	>4	>4	>4
Trimethoprim-sulfamethoxazole	>4	>4	>4	>4
Colistin	0.12	1	0.12	≤0.06
Amikacin	8	8	4	8
Tobramycin	>8	>8	>8	>8

a The percentages of DNA sequence reads displaying mutations on *bla*_KPC-2_ leading to the alteration described are shown in brackets.

Both isolates carried *bla*_KPC-2_ identical to the reference sequence (GenBank accession no. AY034847.1) when PCR amplification followed by Sanger sequencing was performed. The investigation of additional β-lactam resistance mechanisms in the follow-up isolate revealed highly elevated expression of *acrA* in the follow-up isolate that was >1,000-fold (relative quantification value [RQ] of 1,189.0 and minimum to maximum RQ of 795.6 to 1,776.9) that of the susceptible C. freundii control strain, but this increase was only 2.5-fold greater than the expression of this gene in the initial isolate from the same patient. The expression of *ompC* was similar to that of the susceptible control strain, and the initial isolate from the same patient and *ompF* could not be amplified in the expression assay.

Due to the lack of resistance mechanisms that could explain the increase in the ceftazidime-avibactam MIC values, the 2 clinical isolates were submitted for whole-genome sequencing (WGS). OMP sequences were analyzed, and OmpF was found to be disrupted in both clinical isolates. OmpC had no alterations compared to a susceptible control. Single nucleotide polymorphism (SNP) analysis showed minor alterations in genes that have not been previously associated with β-lactam resistance (Table 2).

**TABLE 2:** Single nucleotide polymorphism (SNP) analysis of the *C. freundii* isolates carrying mutated *bla*_KPC-2_

Isolate gene and/or gene description	Reference nucleotide accession no.	% of gene identity of parent to reference	% of reads with SNP	Nucleotide change	Protein accession no.	Amino acid change
Follow-up isolate carrying *blac*_KPC-2_-D179Y						
*bla*_KPC-2_	LT992437.1	100	11	G532T	SPM07605.1	D179Y
Two-component system sensor histidine kinase EnvZ	CP022151.1	100	100	T434G	ASG42425.1	V145G
Hypothetical protein	CP022151.1	99	100	G101T	ASG43191.1	G34V
Glyoxylate carboligase	CP022151.1	100	100	T1292G	ASG45610.1	V431G

Isolate passaged on ceftazidime-avibactam at fixed concn of 4 μg/ml of inhibitor (*bla*_KPC-2_-D176Y)						
*bla*_KPC-2_	LT992437.1	100	80	G523T	SPM07605.1	D176Y

Isolate passaged on ceftazidime-avibactam at 4:1 ratio (*bla*_KPC-2_-R164S + P174L)						
*bla*_KPC-2_	LT992437.1	100	80	C518T	SPM07605.1	P174L
	LT992437.1	100	82	C487A	SPM07605.1	R164S
Hypothetical protein	CP022142.1	96	100	C213T	ASG77181.1	T71M
16S rRNA	CP022151.1	100	88	T472C		

### Passaging, cloning, and characterization of passaged isolates.

The initial patient isolate was submitted to ceftazidime-avibactam passaging experiments in an attempt to recreate the genotype observed in the follow-up patient isolate. After being passaged for 7 days, colonies growing in 32 µg/ml ceftazidime with avibactam at a fixed concentration of 4 µg/ml or at a 4:1 ratio were tested for susceptibility against a panel of β-lactams and were submitted for WGS. The isolates obtained after passaging were highly resistant to β-lactam agents, including carbapenems (Table 1). The sequence analysis and SNP comparison with the initial clinical isolate demonstrated that approximately 78% of the sequence reads of the *bla*_KPC-2_ had the alteration D176Y (*bla*_KPC-2_-D176Y) for the isolated passaged on a fixed concentration of 4 µg/ml, and 82% of the *bla*_KPC-2_ reads had R164S plus P147L substitutions (*bla*_KPC-2_-R164S + P147L) for the 4:1 ratio passaging. These isolates were highly resistant to all β-lactams tested. Prompted by this finding, a thorough analysis of the WGS data of the clinical follow-up isolate was performed and revealed 11% of *bla*_KPC-2_ reads with the alteration D179Y (*bla*_KPC-2_-D179Y, currently named *bla*_KPC-33_; GenBank accession no. NG_056170), which has been associated with elevated ceftazidime-avibactam MIC values in *bla*_KPC-2_ and *bla*_KPC-3_ (6, 8).

To evaluate the stability of the genotypes, 20 colonies obtained from the C. freundii follow-up patient isolate and the isolate passaged with ceftazidime-avibactam at a fixed concentration of 4 µg/ml and purity streaked were streaked on medium without selection, and the colonies were susceptibility tested. For the clinical isolate, 13 of the 20 colonies displayed ceftazidime-avibactam MIC values of ≥16 µg/ml, but 7 had MIC values of 1 to 4 µg/ml for this combination. Sequencing confirmed that ceftazidime-avibactam-susceptible colonies had only the wild-type *bla*_KPC-2_, whereas the colonies exhibiting high ceftazidime-avibactam MIC results had reads displaying the mutation D179Y. For the passaged isolates, 12 of the 20 colonies were resistant to ceftazidime-avibactam, but 8 had reverted to susceptible MIC values for this combination and wild-type *bla*_KPC-2_ sequences. The reversion of phenotype confirms that these colonies might harbor mutated *bla*_KPC-2_ encoding ceftazidime-avibactam resistance and the wild-type version of the gene encoding resistance to meropenem, as seen in the WGS reads.

The impact of the mutations observed was assessed by creating 6 plasmid constructs carrying the *bla*_KPC-2_ gene and genes encoding KPC-2 with alterations D179Y, D176Y, R164S, P147L, and R164S plus P147L. Escherichia coli TOP10 strains carrying the recombinant plasmids were susceptibility tested (Table 3). The MIC values for ceftazidime increased 2- to 8-fold, and those for ceftazidime-avibactam increased 8- to 32-fold for KPC-2 variants. The higher increases were observed for D179Y, D176Y, and P164S plus P174L. The constructs carrying the latter substitutions alone (R164S or P147L) had lower MIC values for ceftazidime-avibactam (2 µg/ml), but the same or slightly higher MIC values compared to the construct producing wild-type KPC-2. The MIC values for ceftriaxone and cefepime alone were 4- to 8-fold lower for the KPC-2 mutants than that of the construct producing wild-type KPC-2. When these cephalosporins were tested with a fixed concentration of 4 µg/ml of avibactam, the MIC values were similar to that for the wild-type KPC-2. A similar effect was noted for aztreonam, which had activity restored for all constructs carrying KPC-2 from 2 to >16 µg/ml to 0.12 and 0.25 µg/ml by adding 4 µg/ml of avibactam (Table 3). As observed by other authors, the MIC values for imipenem and meropenem alone were lower for the mutated KPC-2, and the MIC values were lowered by adding avibactam. Piperacillin MIC values were unchanged with KPC-2 constructs, and avibactam had a similar effect for all recombinant isolates when tested with this agent.

**TABLE 3:** Susceptibility profiles of *E. coli* TOP10 pTRC-*bla*_KPC-2_ constructs

Antimicrobial agent^*a*^	MIC (µg/ml) for E. coli TOP10						
	No plasmid	*bla*_KPC-2_ WT	*bla*_KPC-2_-D179Y	*bla*_KPC-2_-D176Y	*bla*_KPC-2_-R164S + P174L	*bla*_KPC-2_-R164S	*bla*_KPC-2_-P174L
Ceftazidime	≤0.25	16	128	64	128	64	32
Ceftazidime-avibactam	≤0.25	≤0.25	8	8	8	2	2
Ceftriaxone	≤0.03	64	8	4	8	16	16
Ceftriaxone-avibactam	≤0.03	≤0.03	0.12	0.25	0.25	0.12	0.12
Cefepime	≤0.03	8	1	1	1	2	2
Cefepime-avibactam	≤0.03	≤0.03	0.06	0.06	0.12	0.06	0.06
Piperacillin	8	>128	>128	128	128	>128	>128
Piperacillin-avibactam	4	8	16	8	16	16	16
Imipenem	0.25	4	0.25	0.5	0.25	1	2
Imipenem-avibactam	0.12	0.12	0.12	0.12	0.12	0.12	0.25
Meropenem	≤0.015	1	0.03	0.03	0.06	0.25	0.25
Meropenem-avibactam	≤0.015	≤0.015	≤0.015	≤0.015	≤0.015	≤0.015	≤0.015
Aztreonam	0.12	>16	2	8	2	>16	>16
Aztreonam-avibactam	0.12	0.25	0.25	0.25	0.25	0.12	0.12

a Avibactam was tested at a fixed concentration of 4 µg/ml in all cases.

## DISCUSSION

Similar to other literature reports about ceftazidime-avibactam resistance developing during treatment, we observed the increase in ceftazidime-avibactam MIC values from 4 to 64 µg/ml in a C. freundii isolate carrying *bla*_KPC-2_. Our investigation showed that both clinical isolates had a highly elevated expression of AcrAB-TolC compared to a C. freundii control strain susceptible to all β-lactams. This mechanism has been recently associated with an increased ceftazidime-avibactam MIC value of 16 µg/ml (5); however, since both patient isolates had similar expression of this efflux system, the hyperexpression of this gene was unlikely to cause the increase in the ceftazidime-avibactam MIC during treatment. The elevated expression of AcrAB-TolC and OMP alterations could, however, explain the elevated MIC result of 4 µg/ml in the initial C. freundii isolate.

The initial results from the patient follow-up C. freundii isolate did not indicate a mutation on *bla*_KPC-2_ since the gene carrying these alterations usually encodes an extended-spectrum β-lactamase-like phenotype and susceptibility to carbapenems, and the follow-up isolate evaluated in this study was resistant to all carbapenems tested. WGS analysis of the isolates obtained after ceftazidime-avibactam passaging experiments exhibited a percentage of the *bla*_KPC-2_ sequence reads with mutations leading to amino acid substitutions D176Y or R164S plus P174L, which are located in the Ω-loop (11) and were demonstrated to encode ceftazidime and ceftazidime-avibactam resistance. Further investigations on the follow-up C. freundii patient isolate showed that a small percentage of the *bla*_KPC-2_ sequencing reads had mutations leading to the D179Y alteration, which has been previously reported to increase ceftazidime hydrolysis and decrease inhibition by avibactam (8). The low percentage (11%) of reads was in the initial WGS analysis but was unnoticed.

Sequencing reads of the wild-type and mutated *bla*_KPC-2_ genes were present in the clinical and passaged isolate experiments that generated high MIC values to all β-lactam agents. Subculturing these isolates in the absence of selective pressure demonstrated that part of the population reverted to wild-type *bla*_KPC-2_ sequences to the detriment of the mutant reads. However, we could not separate colonies carrying the wild-type and the D179Y mutant from the C. freundii clinical isolate, despite multiple attempts.

Mutations of the Ω-loop of various β-lactamase enzymes increased the hydrolysis for ceftazidime, and the KPC-2 variants carrying D179Y alterations were able to trap the ceftazidime molecule for longer periods and avoid binding of avibactam (7, 8). This effect was not observed with other β-lactams tested, and our results demonstrate that avibactam was still able to inhibit the KPC-2 mutants harboring D179Y, D176Y, and R164S plus P174L when paired with ceftriaxone, cefepime, aztreonam, imipenem, and meropenem.

Our findings implicate that once the ceftazidime-avibactam selective pressure is removed, the population might revert to a majority of the original genotype and again display elevated MIC values for other β-lactams, including carbapenems, which means that β-lactams alone might not be used, as suggested by others. Instead, addition of another β-lactam such as cefepime, aztreonam, or a carbapenem might be more prudent to make sure that coverage is provided for both populations.

Other antimicrobial agents displaying activity against KPC-producing isolates have been approved or are in late-stage development, but as shown with the experience of ceftazidime-avibactam, only clinical use will reveal the advantages and limitations of each of them; however, at least now more options exist to treat these troublesome organisms.

## MATERIALS AND METHODS

### Bacterial strains.

Two C. freundii clinical isolates recovered from blood and abdominal fluid from a patient hospitalized in South Carolina in September 2015 were submitted to a reference laboratory for confirmation of susceptibility results. A timeline of the infection and the antibiotics used is presented in Fig. 1. Bacterial identification was confirmed by matrix-assisted laser desorption ionization–time of flight mass spectrometry (MALDI-TOF MS [Biotyper; Bruker Daltonics, Billerica, MA]). Isolates were susceptibility tested using the broth microdilution method as described by CLSI (12). Ceftazidime-avibactam was also tested using Etest strips (bioMérieux, St. Louis, MO) following the manufacturer’s instructions and disk diffusion according to CLSI guidelines (13). The categorical interpretations for all antimicrobial agents were those found in M100 (9, 10), and quality control (QC) testing was performed using E. coli ATCC 25922 and Pseudomonas aeruginosa ATCC 27853. All QC results were within specified ranges as published in CLSI documents.

### Molecular typing.

C. freundii clinical isolates were epidemiologically typed by pulsed-field gel electrophoresis. Genomic DNA prepared in agarose blocks and digested with SpeI (New England Biolabs, Beverly, MA) was resolved in the CHEF-DR II contour-clamped homogeneous electric field apparatus (Bio-Rad, Richmond, CA). Results were analyzed by GelCompar II software (Applied Math, Kortrijk, Belgium). Percentages of similarity were identified on a dendrogram derived from the unweighted pair group method using arithmetic averages and based on Dice coefficients. Band position tolerance and optimization were set at 1.2% and 0.5%, respectively.

### Evaluation of β-lactam resistance mechanisms.

C. freundii clinical isolates were initially screened for the presence of *bla*_KPC_, *bla*_SME_, *bla*_GES_, *bla*_NMC-A_, *bla*_IMI_, *bla*_IMP_, *bla*_VIM_, and *bla*_NDM_ as previously described (14). The amplicons generated were sequenced on both strands; nucleotide and deduced amino acid sequences were analyzed using the Lasergene software package (DNASTAR, Madison, WI).

The relative expression levels of *ompC*, *ompF*, and *acrA* (AcrAB-TolC) for the C. freundii clinical isolates were determined by quantitative real-time PCR using DNA-free RNA preparations. RNA extraction and treatment and relative quantification of target genes were performed as previously described (15). The expression rates were normalized using *rpsL*, and the expression results were compared to those from a C. freundii clinical isolate susceptible to β-lactams and other antimicrobial classes. Transcription levels were considered significantly different if at least a 10-fold increase was observed for *acrA* and a decrease for the OMP genes was noted compared with the control.

### Serial passaging.

The initial C. freundii patient isolate was serially passaged on ceftazidime concentrations of 1 to 128 µg/ml with avibactam at either a fixed concentration of 4 µg/ml or a 4:1 ratio. The parent strain was grown in 4-ml volumes of cation-adjusted Mueller-Hinton broth using an initial starting inoculum of approximately 1 × 10^6^ CFU/ml. Subsequent daily passages utilized 20 µl of the tube 1 log_2_ dilution step below the MIC from the previous day’s serial passage. This process was repeated for 7 daily transfers. Tubes were incubated at 35°C in ambient atmosphere without shaking and examined for growth at 24 h. Following passaging, cultures were streaked onto a Mueller-Hinton agar plate for isolated colonies. One colony per passage was then selected for confirmatory broth microdilution susceptibility testing and WGS. To evaluate the stability of the genotypes, 20 colonies of the C. freundii follow-up isolate and the mutant generated on ceftazidime-avibactam at a fixed concentration of 4 µg/ml were subcultured twice in medium without selection and submitted to susceptibility testing. Selected colonies were sequenced.

### WGS analysis.

The two C. freundii clinical isolates—one isolate obtained from the fixed 4-µg/ml passaging and one isolate from the 4:1 ratio passaging—were submitted to WGS. Total genomic DNA was used as input material for library construction prepared using the Nextera XT library construction protocol and index kit (Illumina, San Diego, CA) following the manufacturer’s instructions. Sequencing was performed on a MiSeq sequencer (Illumina). BWA v.0.7.12-r1034 was used to map the initial reads onto an input reference genome. The results were sorted and indexed with SAMtools v.1.4. Picard v1.103 was used to mark duplicates. Freebayes v.1.1.0 was used to call variants, and the resulting VCF file was used to generate a consensus genome from the portion of the reads that mapped to the input reference genome. The VCF file was also used to adjust positions of the input annotation file so that gene boundaries were retained for this generated initial genome. All unmapped reads were then *de novo* assembled using SPAdes v.3.10.0. The resulting scaffolds were subjected to a BLAST search. The scaffolds were then added to the initial genome as new chromosomes; their annotations were appended to the adjusted annotation file. The follow-up reads were then mapped to the initial genome using BWA, and the results were sorted and indexed using SAMtools. The duplicates were marked using Picard, and the variations were called with FreeBayes. The resulting VCF file was then processed by SNPeff v.4.3K to annotate each variation. The resulting output was filtered for each variant, requiring a quality score of ≥40, alternative haplotype of ≥77%, and read depth of ≥5. Variants were then analyzed visually using SeqMan NGen v.14.0 (DNASTAR).

### Cloning *bla*_KPC-2_ mutants.

Mutant alleles of *bla*_KPC-2_ and the pTRC99a promoter were synthesized by Integrated DNA Technologies (Coralville, IA) and cloned into the pUCIDT-Kan vector containing a pMB1 origin and a kanamycin resistance gene. The plasmids were transformed into E. coli TOP10 cells (Invitrogen, Carlsbad, CA) and selected on nutrient agar plates containing 25 µg/ml kanamycin. Following a purity streak with the same selection, isolates were susceptibility tested against β-lactams alone and with a fixed concentration of 4 µg/ml of avibactam. IPTG (isopropyl-β-d-thiogalactopyranoside) was not included in the testing medium but rather relied on leaky expression of the pTRC99a promoter for production of the mutant KPC-2 proteins.
